# Global epidemiology of podoconiosis: A systematic review

**DOI:** 10.1371/journal.pntd.0006324

**Published:** 2018-03-01

**Authors:** Kebede Deribe, Jorge Cano, Mei L. Trueba, Melanie J. Newport, Gail Davey

**Affiliations:** 1 Wellcome Trust Brighton and Sussex Centre for Global Health Research, Brighton and Sussex Medical School, Brighton, United Kingdom; 2 School of Public Health, Addis Ababa University, Addis Ababa, Ethiopia; 3 Department of Disease Control, London School of Hygiene & Tropical Medicine, London, United Kingdom; University of Tennessee, UNITED STATES

## Abstract

**Background:**

Podoconiosis is one of the few diseases that could potentially be eliminated within one generation. Nonetheless, the global distribution of the disease remains largely unknown. The global atlas of podoconiosis was conceived to define the epidemiology and distribution of podoconiosis through dedicated surveys and assembling the available epidemiological data.

**Methods:**

We have synthesized the published literature on the epidemiology of podoconiosis. Through systematic searches in SCOPUS and MEDLINE from inception to February 14, 2018, we identified observational and population-based studies reporting podoconiosis. To establish existence of podoconiosis, we used case reports and presence data. For a study to be included in the prevalence synthesis, it needed to be a population-based survey that involved all residents within a specific area. Studies that did not report original data were excluded. We undertook descriptive analyses of the extracted data. This study is registered with PROSPERO, number CRD42018084959.

**Results:**

We identified 3,260 records, of which 27 studies met the inclusion criteria. Podoconiosis was described to exist or be endemic in 32 countries, 18 from the African Region, 3 from Asia and 11 from Latin America. Overall, podoconiosis prevalence ranged from 0·10% to 8.08%, was highest in the African region, and was substantially higher in adults than in children and adolescents. The highest reported prevalence values were in Africa (8.08% in Cameroon, 7.45% in Ethiopia, 4.52% in Uganda, 3.87% in Kenya and 2.51% in Tanzania). In India, a single prevalence of 0.21% was recorded from Manipur, Mizoram and Rajasthan states. None of the Latin American countries reported prevalence data.

**Conclusion:**

Our data suggest that podoconiosis is more widespread in the African Region than in the rest of the regions, although this could be related to the fact that most podoconiosis epidemiological research has been focused in the African continent. The assembled dataset confirms that comprehensive podoconiosis control strategies such as promotion of footwear and personal hygiene are urgently needed in endemic parts of Africa. Mapping, active surveillance and a systematic approach to the monitoring of disease burden must accompany the implementation of podoconiosis control activities.

## Author summary

Podoconiosis is one of the Neglected Tropical Diseases, and causes painful and massive swelling of the lower legs. Despite the importance of the disease, its global distribution and epidemiology are poorly understood. We conducted a systematic review to assess the global epidemiology of podoconiosis. We found that podoconiosis was described to exist or be endemic in 32 countries, 18 from Africa, 3 from Asia and 11 from Latin America. Almost all prevalence data recorded corresponded to the African region. None of the Latin American countries reported prevalence data, although some countries are suspected to be endemic. We recommend that comprehensive podoconiosis control strategies such as promotion of footwear and personal hygiene are urgently needed in endemic countries in the African Region. Mapping, active surveillance and a systematic approach to the monitoring of disease burden must accompany the implementation of podoconiosis control activities.

## Introduction

Podoconiosis is a neglected tropical disease caused by exposure to red clay soil [1, 2]. The disease results from a complex interaction between genes and the environment occurring over many years. Mineral particles from the soil penetrate the skin and are taken up by macrophages in the lymphatic system which causes inflammation and fibrosis of the vessel lumen leading to blockage of the lymphatic drainage. This results in oedematous feet and legs and subsequently progresses to elephantiasis, including nodular skin changes [3]. These changes are themselves disabling, and painful intermittent acute inflammatory episodes cause further debility[4].

Current global estimates suggest that there are 4 million cases of podoconiosis in Africa, parts of Latin America and South East Asia[5, 6]. In 2011, the World Health Organization (WHO) included podoconiosis in the list of neglected tropical diseases (NTDs)[7]. Intervention includes prevention through consistent use of footwear starting from an early age, regular foot hygiene and covering housing floors. For those with the disease, simple lymphedema management consisting of foot hygiene, foot care, wound care, compression, exercises and elevation, treatment of acute attacks and use of shoes and socks to reduce further exposure to the irritant soil is recommended [8].

Despite increased global interest in podoconiosis, the global epidemiology of the disease is largely uncertain [9]. This is partly due to the absence of accurate and easy-to-use diagnostic tools such as a point-of-care diagnostic test. Currently, diagnosis is clinical and based on exclusion of other potential causes of lymphedema in the tropics, mostly lymphatic filariasis but also certain forms of leprosy, Milroy syndrome and heart or liver failure, for example. [10]. Nonetheless, in endemic areas trained health workers can easily identify the disease [11].

Although high prevalence of podoconiosis has been reported intermittently across a range of settings, it has never been prioritized either in intervention or research programmes. This may be due to the lack of resources for new health initiatives, which is a common problem in the low-income tropical countries in which this disease is present. Only two countries (Ethiopia and Rwanda) report podoconiosis within their routine health management information systems [12]. In other well-known or suspected endemic countries, the existence of podoconiosis is based on cross-sectional surveys, case reports and presence reports intended to ascertain the disease presence and burden in specific areas [13, 14].Therefore, to understand the global distribution of podoconiosis, compiling the existing evidence is of utmost importance. Keeping these repositories of epidemiological data up-to-date will be relevant for further monitoring and evaluation of interventions put in place, as similar experiences have come to demonstrate [15, 16]. Price’s monograph published in 1990 [3] is the first attempt to review the distribution of podoconiosis globally. Despite being a comprehensive work, it did not follow current systematic guidelines on data assembling and survey selection.

Building upon this first experience, we have conducted a systematic review of studies reporting prevalence data of podoconiosis across major libraries of scientific literature and through searches on gray literature (i.e. unpublished reports, monographies, etc.). In addition, we undertook a descriptive analysis of the extracted data.

## Materials and methods

### Search strategy and selection criteria

**Fig 1:**
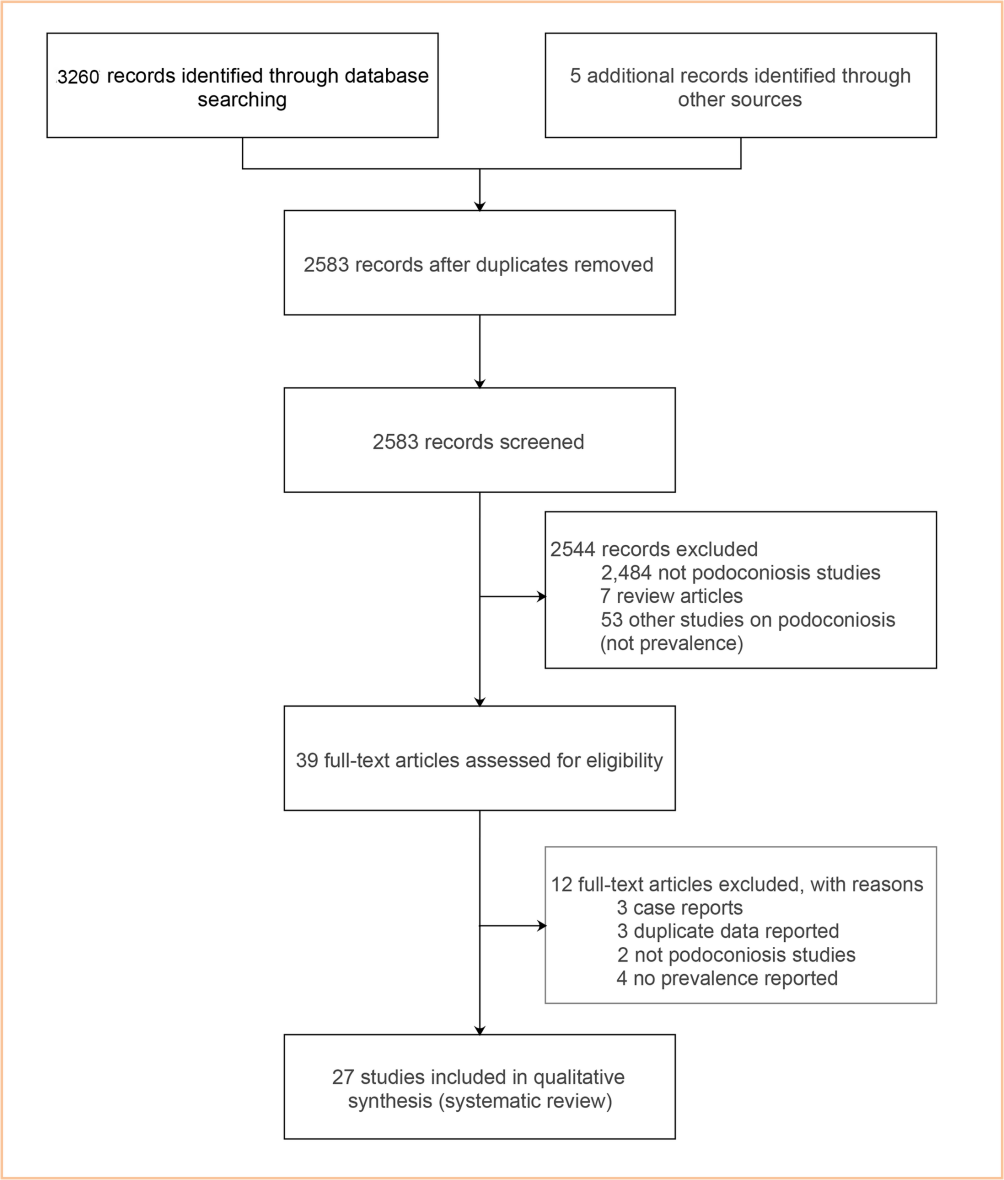
Selection of eligible studies.

We searched for studies that reported the epidemiology of podoconiosis, using a systematic review approach that followed the Preferred Reporting Items for Systematic Reviews and Meta-Analyses (PRISMA) [17] (Fig 1). We searched MEDLINE from inception (coverage from 1950) to 14 February 2018 and SCOPUS (from 1966 to 14 February, 2018) for all relevant studies that examined podoconiosis prevalence. We used the following search terms; ‘podoconiosis’ OR ‘mossy foot’ OR ‘non-filarial elephantiasis’ combined with ‘prevalence’, ‘epidemiology’, ‘public health’, ‘population’ (see S1 Text: Supplementary File). No time, geographical area or language limits were applied. We hand-searched the reference lists of all recovered documents for additional references. Abstracts of all reports were read, and full papers retrieved for those appearing to fulfill selection criteria, as it is detailed below (Fig 1). This analysis is reported using recommended criteria (see S1 Checklist).

We searched the gray literature by seeking reports not published in peer reviewed journals through contacting experts, a search of conference abstracts and reviewing Price’s monograph [12].

### Study selection

We screened search results first by title and abstract and then by full text. We disregarded abstracts in the initial screen if they were not observational studies and did not investigate the epidemiology of podoconiosis. We also excluded studies that did not report original data (e.g. review articles). Abstracts reporting observational studies and/or focusing on podoconiosis were eligible for full-text review. Population-based articles were independently considered for inclusion in the review if the studies reported prevalence of podoconiosis or contained adequate information to calculate prevalence. By ‘population-based’ articles we meant studies which involved all residents within a specific area and in which the studied population was representative of that area. We excluded studies based on data recorded in health facilities. When the same data were reported in two or more publications, we selected the most comprehensive source. Lastly, we identified papers outside the search strategy using expert knowledge of active studies and contacting experts. When possible, we contacted authors to provide data not presented in their reports.

### Study eligibility and quality assessment

All studies reporting the prevalence of podoconiosis at community level were included. Case reports and presence data were used to identify countries which are currently endemic or those previously endemic. All titles and abstracts of the identified studies were assessed for relevance for the review by two authors using a third author to resolve any discordance. Relevant full text articles were retrieved and checked by two authors (KD and JC) independent of each other, with reference to GD in case of differences. Non-English language papers were translated using Google Translate (Google, Mountain View, CA, USA) or by colleagues proficient in the language in question.

The quality of the studies was assessed by two authors, with disagreement resolved by referral to a third author. The quality of the studies in the review was assessed against four criteria: definition of sampling frame, response rate, quality of podoconiosis assessment and reporting bias (S1 Text. Supplementary File). We adopted the quality assessment framework set by the Newcastle–Ottawa Scale [18]. The four criteria were assessed on a three-point numerical scale (0, 1, or 2) and involved evaluation of the sampling frame, attrition rate (through assessment of non-response), the measurement criteria (through evaluation of podoconiosis diagnosis criteria), and assessment of reporting bias (by showing how well the study covered reporting of age and other key population descriptions).

### Data extraction and synthesis

After searching for the data in MEDLINE all the references were exported to EndNote X7(endnote.com).A standardized Microsoft Excel data extraction form was developed and used to record the following information for all qualifying studies: study ID, author, title, journal, the year of study, study design, geographical location (area and country), sample size, number of cases, descriptive case data (e.g., age, sex) and diagnostic method.

We collected data on prevalence with 95% confidence intervals (CIs). Data were extracted by two authors (KD, JC) with any disagreements referred to a third author (GD) for resolution. We then exported the data to STATA statistical suite (Version 15.0, StataCorp LP, College Station, Texas, USA) for further analysis.

We undertook descriptive analyses of the extracted data. Prevalence estimates are presented with 95% CIs for those studies with reported cases of podoconiosis and a reported sample size. All analyses were conducted within the STATA statistical suite.

We downloaded the base map of the global administrative areas from the GADM (www.gadm.org) [19]. Point maps displaying the distribution of data points and average podoconiosis prevalence were produced using ArcGIS Desktop v10.3 (Environmental Systems Research Institute Inc., Redlands CA, USA).

This systematic review is registered in the PROSPERO International Prospective Register of systematic reviews, registration number CRD42018084959 (https://www.crd.york.ac.uk/prospero/display_record.php?RecordID=84959).

## Results

Our systematic review identified 3,260 records through the database search and 5 additional records through the manual search for gray literature. After duplicates were removed, 2,583 studies were screened based on their titles and abstracts. Thirty-nine articles were assessed for eligibility. As a result of this assessment, 27 studies met the inclusion criteria and were included in the systematic review [3, 4, 14, 20–49], see Fig 1. The list of included studies is provided in Table 1.

**Table 1:** Podoconiosis prevalence studies included in the review.

Study, study or publication year	Year of study	Country	Location	Sampling method	Case ascertainment	Case (sample size)	Prevalence, % (95%CI)
Africa							
Price, 1976[42]	1976	Burundi & Rwanda	Nationwide	Market survey	Observation of frank lymphoedema	Burundi 61 (6,156) Rwanda 128 (20,446)	0.99 (0.77–1.27) 0.63 (0.53–0.75)
Wanji et al 2008[49]	2006	Cameroon	North West province, Ndop and Tubah	Community based	Clinical, parasitological and entomological	66 (817)	8.08 (6.21–9.95)
Cho-Ngwa et al., 2009[22]	2003	Cameroon	Bambui Health District of NW Cameroon	Community based	Clinical and parasitological	16 (301)	5.32 (3.30–8.46)
Wanji et al.,2016[13]	2016	Cameroon	Bafut, Bamenda, Batibo,Mbengwi, Ndop and Tubah	Community based	Clinical	1069 (56,479)	1.90 (1.80–2.00)
Deribe et al.,2017[50]	2017	Cameroon	Nationwide	Community based	Clinical, parasitological, and molecular	52 (10,178)	0.50 (0.40–0.70)
Price 1990[3]	1988	Equatorial Guinea	Bioko Island Equatorial Guinea	Community based	Clinical	26 (3577)	0.73 (0.50–1.07)
Bekele et al.,2016[4]	2015	Ethiopia	Wayu Tuka woreda	Community based	Clinical	1,197 (39,256)	3.05 (2.90–3.20)
Deribe et al, 2015[29]	2013	Ethiopia	Nationwide	Community based	Clinical and ICT	5,253 (129,959)	4.04 (3.93–4.15)
Tekola Ayele et al, 2013[48]	2011	Ethiopia	Bedele Zuria district	Community based	Clinical	379 (6,710)	5.65 (5.12–6.23)
Molla et al, 2012[38]	2011	Ethiopia	Debre Eliyas and Dembecha districts	Community based	Clinical	1,704 (51,017)	3.34 (3.19–3.50)
Geshere Oli et al, 2012[33]	2012	Ethiopia	Midakegn district	Community based	Clinical, parasitological	123 (1,656)	7.43 (6.26–8.79)
Alemu et al, 2011[20]	2011	Ethiopia	Gulliso woreda	Community based	Clinical	1,935 (69,465)	2.79 (2.67–2.92)
Desta et al, 2007[30]	2001	Ethiopia	Wolaitta Zone	Community based	Clinical	1890 (33,678)	5.46 (5.21–5.71)
Birrie et al., 1997 [21]	1997	Ethiopia	Pawe settlement area	Community based	Clinical, parasitological	68 (1,900)	3.58 (2.83–4.51)
Frommel et al, 1993 [32]	1993	Ethiopia	Ocholo	Community based	Clinical	153 (3,022)	5.06 (4.33–5.90)
Kloos et al., 1992 [35]	1992	Ethiopia	Gera & Didessa, Western Ethiopia	Community based	Clinical	31 (416)	7.45 (5.30–10.52)
Mengistu et al., 1987 [37]	1987	Ethiopia	Ocholo Gamo Gofa	Community based	Clinical	146 (2,689)	5.43 (4.64–6.35)
Price,1974 [51]	1974	Ethiopia	Wolaitta Zone, Wajifo, Shenoe and Alaba	Market survey	Observation of frank lymphoedema	1781 (43,573)	4.09 (3.91–4.28)
Oomen, 1969 [40]	1969	Ethiopia	Nationwide	Market survey	Observation of frank lymphoedema	6770 (247,908)	2.73 (2.67–2.79)
Crivelli, 1986 [26]	1986	Kenya	Nyambene Range	Community based	Clinical and parasitological	105 (2,711)	3.87 (3.21–4.66)
Muli et al, 2017 [52]	2017	Kenya	Mt. Longonot region in Nakuru County	Community based	Clinical, parasitological and molecular	13 (385)	3.40(1.80, 5.70)
Ruiz 1994 [45]	1988	Sao Tome & Principe	Districts of Cantagalo and Lemba	Community based	Clinical and pathological	11 (1,200)	0.92 (0.52–1.64)
Onapa et al., 2001 [39]	1998	Uganda	Kapchorwa District	Community based	Clinical, parasitological and entomological	26 (575)	4.52 (3.10–6.54)
Kihembo et al., 2017 [53]	2015	Uganda	Kamwenge District, western Uganda	Community based	Clinical and ICT	52 (51,553)	0.10(0.10–0.10)
De Meira et al, 1947 [28]	1947	Cape Verde	Island of S. Nicolau	Community based survey	Clinical, parasitological and histological	21 (7,000)	0.30 (0.20–0.46)
Jordan et al., 1956 [34]	1956	Tanzania	Bukoba, Biharamulo, Ngara, and Kibondo Districts.	Community based survey	Clinical, antigen based	12(475)	2.51(1.10–3.92)
Asia							
Russel et al., 1983 [46]	1974–1982	India	Imphal, Aizawal & Bikaner	Community based survey	Clinical and parasitological	9 (4,214)	0.21 (0.11–0.40)

Overall, the quality of the studies included in the review was poor (S1 Text: Supplementary File). The commonest issue with study quality was the lack of detail on the diagnosis of podoconiosis. Only three studies met the quality standard in all four criteria, 18 studies described the sampling frame thoroughly, 7 studies provided the response rate, and 16 studies satisfactorily included a report on podoconiosis assessment. The scale of the surveys differed, some focused on an entire province, whereas others focused on randomly selected communities or households. The largest studies were conducted in Africa: in Ethiopia two studies included 247,908 [40] and 129,959 individuals [29], in Rwanda over 20,446 [42], and in Cameroon 10,178 [50]. In this region, the smallest sample size reported in a study was 301 participants in Cameroon [22].

To document the countries with current and historical presence of the disease, we compiled case and presence reports. Overall, 32 countries reported existence of podoconiosis now or previously. For eleven of them, prevalence data were available (Burundi [42], Cameroon, Cape Verde, Equatorial Guinea, Ethiopia, India, Kenya, Rwanda [42], Sao Tome & Principe, Uganda and Tanzania), two countries had case data (Brazil [1 case] and Sudan [28 cases]) [3] and 19 countries reported presence without case data (Angola, Chad, Colombia, Costa Rica, Democratic Republic of Congo, Ecuador, El Salvador, French Guiana, Guatemala, Honduras, Indonesia, Madagascar, Mexico, Mozambique, Niger, Nigeria, Peru, Sri Lanka, and Suriname) [3]. (Table 2)

**Table 2:** Countries reporting existence of podoconiosis.

SN	Country	Evidence included
1	Brazil	Case report [47]
2	Sudan	Case report [54]
3	Angola	Presence report [3]
4	Chad	Presence report [55]
5	Colombia	Presence report [3]
6	Costa Rica	Presence report [3]
7	Democratic Republic of the Congo	Presence report [3]
8	Ecuador	Presence report [3]
9	El Salvador	Presence report [3]
10	French Guiana	Presence report [3]
11	Guatemala	Presence report [3, 56]
12	Honduras	Presence report [36]
13	Indonesia	Presence report [3]
14	Madagascar	Presence report [3]
15	Mexico	Presence report [3, 57]
16	Mozambique	Presence report [3]
17	Niger	Presence report [55]
18	Nigeria	Presence report [3]
19	Peru	Presence report [36]
20	Sri Lanka	Presence report [3]
21	Suriname	Presence report [3]
22	Burundi	Survey data [42]
23	Cameroon	Survey data[13, 22, 49, 50]
24	Cape Verde	Survey data [28]
25	Equatorial Guinea	Survey data [3]
26	Ethiopia	Survey data [4, 20, 21, 29, 33, 35, 37, 38, 40, 48, 58]
27	India	Survey data [46]
28	Kenya	Survey data [26, 52]
29	Rwanda	Survey data [42]
30	Sao Tome and Principe	Survey data [45]
31	Uganda	Survey data [39, 53]
32	United Republic of Tanzania	Survey data [34]

**Fig 2:**
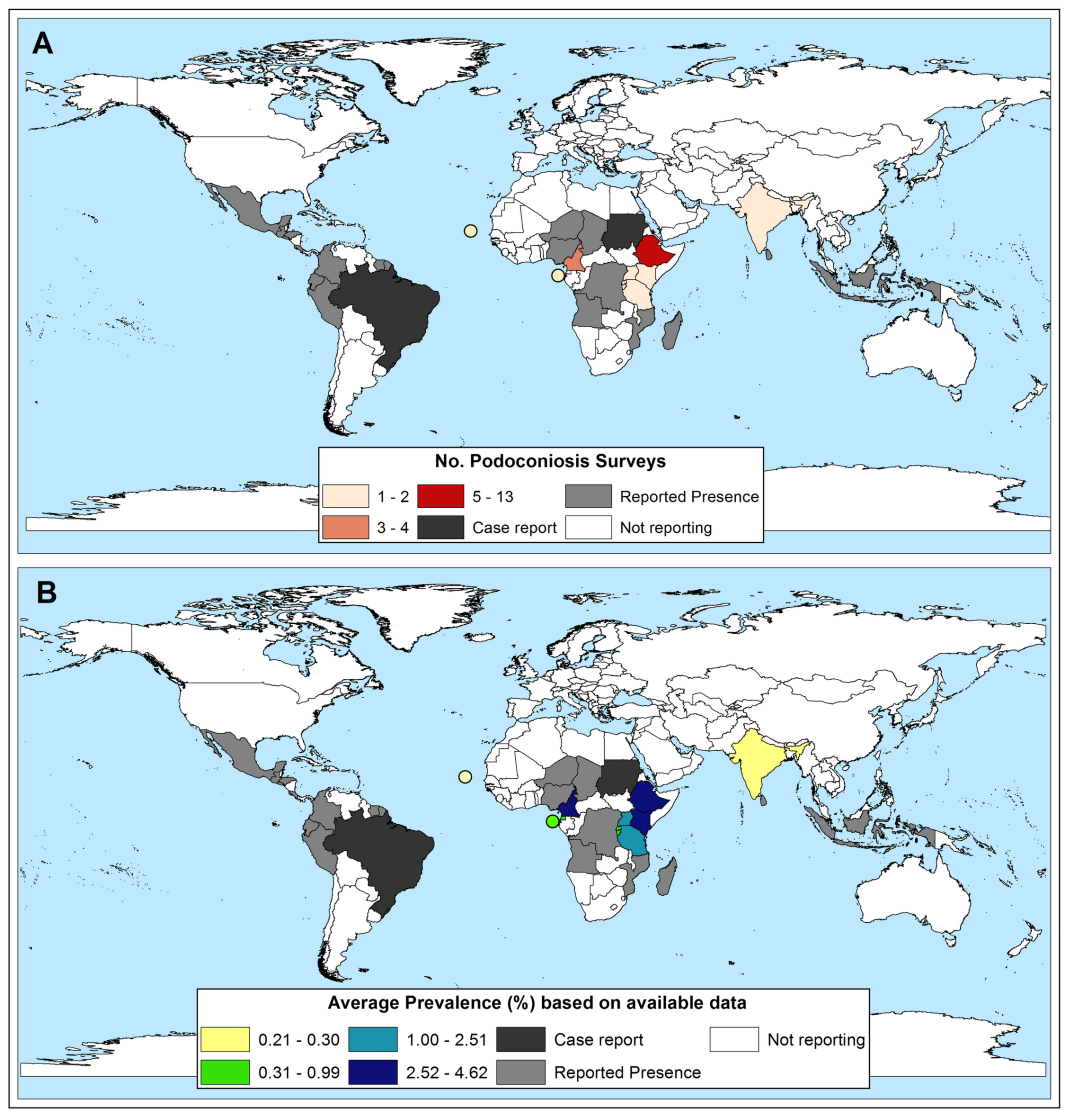
Geographical distribution of A) Surveys included B) Prevalence of podoconiosis. Dots represent various island nations.

Eighteen countries reporting podoconiosis were from Africa, 3 from Asia and 11 from Latin America. All but one of the countries reporting prevalence data were in Africa (10), the exception being India. None of the Latin American countries reported prevalence data, only Brazil reported case data, and the remainder reported observations without specific case data (Fig 2).

The prevalence studies covered 11 countries, ten of them within the African region: Burundi, Cameroon, Cape Verde, Equatorial Guinea, Ethiopia, Kenya, Rwanda, Sao Tome & Principe, Uganda and Tanzania. The remaining study was from three states in India: Manipur, Mizoram and Rajasthan. The prevalence of podoconiosis was recorded in 27 studies and ranged from 0.1% in Uganda [53] to 8.08% in Cameroon [49]. Of the 27 studies reporting prevalence, 13 were conducted in Ethiopia where prevalence ranged from 2.73% to 7.45% [4, 20, 21, 29, 30, 32, 33, 35, 37, 38, 40, 41, 48]. Podoconiosis is widespread in Ethiopia with more than 345 districts endemic for the disease [59]. The national average prevalence is estimated to be 4.0% [29]. Another study reported 1.6 million people living with podoconiosis in Ethiopia with 35 million people at risk of the disease in the country [59, 60]. Cameroon is the other country where complete mapping of the distribution of podoconiosis has been done. The prevalence of podoconiosis in Cameroon ranges from 0.5% to 8.08% [49, 50]. Podoconiosis is widespread in Cameroon and found in every region of the country.

**Fig 3:**
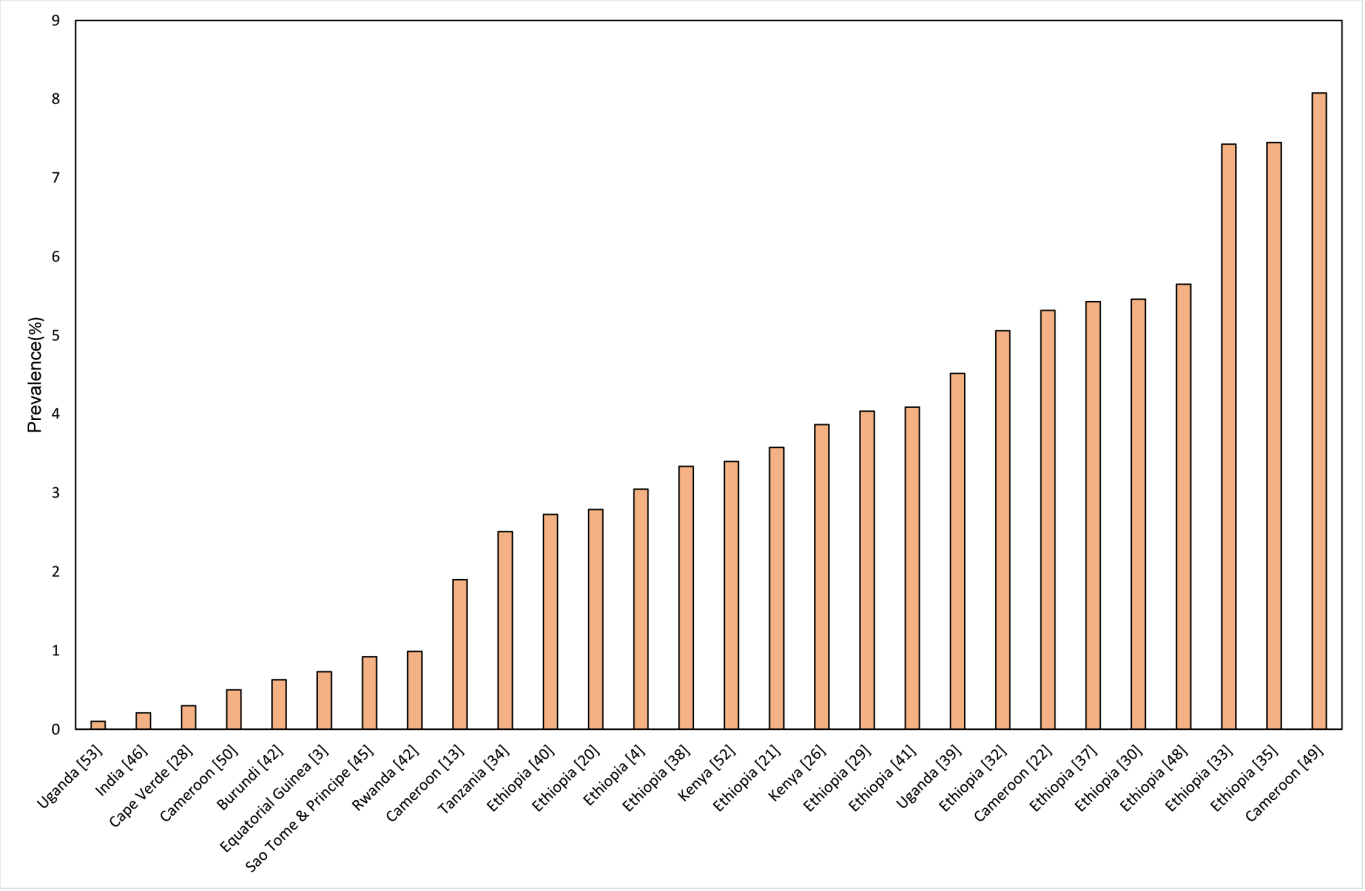
Prevalence of podoconiosis by country.

High prevalence of podoconiosis was also recorded in other countries, including 4.52% in Uganda [39], 3.87% in Kenya [26, 52], and 0.99% in Burundi [42]. In Rwanda, the prevalence of podoconiosis is estimated at 0.63%[42], whereas in Sao Tome & Principe it is 0.92% [45](Fig 3).

**Fig 4:**
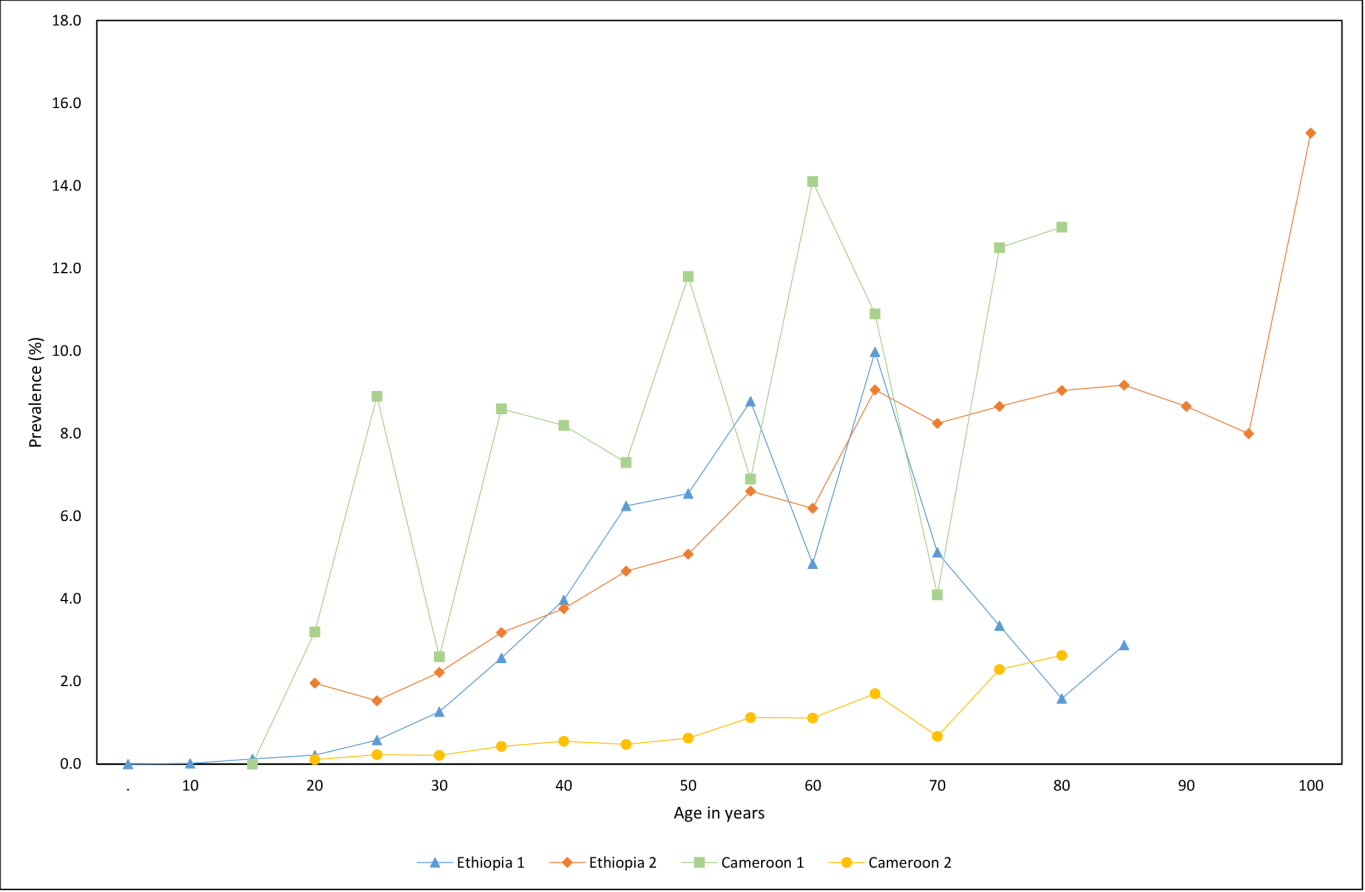
Podoconiosis prevalence by age group in selected studies Ethiopia 1 [4], Ethiopia 2 [29], Cameroon 1 [49] and Cameroon 2 [50].

Overall, 6 studies reported prevalence by age group [4, 29, 32, 39, 49, 50]. The prevalence of podoconiosis was higher among adults than children [4, 32, 39]. Of the three studies including children under 10 years old, two reported 0% prevalence [4, 39] whilst one reported 0.4%[32]. In Ethiopia and Cameroon, the prevalence of podoconiosis increased with age (Fig 4).

## Discussion

Podoconiosis is one of the major causes of tropical lymphedema [2, 61], but its global prevalence remains elusive. Access to updated evidence on the incidence and prevalence of podoconiosis is essential for guiding and informing global, regional, and national health policies. The systematic review presented here has collated all the currently available literature regarding the prevalence of podoconiosis, and included 27 prevalence studies predominantly from Africa. Although the methods and diagnosis of podoconiosis varied from one study to another, the articles included in this review generally indicated high prevalence of podoconiosis.

Based on the available evidence, 32 countries provided data suggesting the existence of podoconiosis currently or historically. This is based on 11 countries with prevalence data, two countries with case reports and 19 countries with reported presence of the disease but no epidemiological data. The interpretation of these findings is very difficult, particularly for the countries where only presence is reported. Although the rates of poverty and environmental conditions are compatible with the existence of podoconiosis, unless information about the presence of the disease is backed by epidemiological surveys or surveillance data, a national or global response to podoconiosis cannot be initiated. This is particularly evident for countries reporting isolated cases, such as Brazil [47], where only one case was reported in the published literature. It is vital to gather more information on the current status of podoconiosis in countries such as Brazil. Previous experience from endemic countries has showed that underreporting of the disease is very common owing to poor awareness and lack of systematic scrutiny by health workers [62]. Hence, the distribution of podoconiosis might reach beyond the countries listed here, as the necessary environmental and socio-economic conditions exist in other areas. While the purpose of the current review is to increase robust surveillance in the listed countries, we would like to emphasize the list here is only guided by the available literature.

Most of the highly-affected countries are in African Region. In this region, we identified prevalence data from 10 countries (Burundi, Cameroon, Cape Verde, Equatorial Guinea, Ethiopia, Kenya, Rwanda, Sao Tome & Principe, Uganda and Tanzania). India is the only country with prevalence data outside of the African Region, and these data are more than three decades old. The prevalence of podoconiosis is particularly high in Cameroon [49], Ethiopia [32, 33, 35] and Uganda [39]. The high prevalence of the disease in these countries could be due to low levels of footwear use and access to water, along with environmental suitability for the occurrence of the disease, namely highland rural areas, with high precipitation and soil typically rich in silt and clay particles [29]. Previous studies have indicated that 24% of the landmass in Ethiopia (where 44% of the population lives) is suitable for the occurrence of podoconiosis [59]. Similarly, in Cameroon the disease is widespread and cases were identified in every region of the country [50].

Our study has some limitations. We did a comprehensive systematic review of the published literature on the prevalence of podoconiosis, but we chose not to do a meta-analysis because of variability between studies. The major issue here is the diagnosis of podoconiosis. Until recently there was no standardized case definition for podoconiosis [63, 64]. Earlier studies used clinical diagnosis of podoconiosis, without clear indication of how other potential causes of lymphedema were excluded. More recent studies have used a combination of clinical, parasitological and molecular techniques to exclude other potential causes of lymphedema [10, 50]. Some recent studies did not outline how the diagnosis of podoconiosis was established. In an attempt to standardize podoconiosis surveys, we have provided guidance based on consensus and evidence-based diagnosis of podoconiosis [65]. The other limitation of the collated studies is that surveys tended to be conducted in areas where podoconiosis was suspected to be an important problem, which may lead to overestimation of the prevalence. Nonetheless there are exceptions where nationwide surveys were conducted, including in Burundi [42], Cameroon [50], Ethiopia [29] and Rwanda [42]. Most of the data included in this review are from the African Region as data are scarce for other regions of the world. Countries in which podoconiosis is less prevalent may have not reported the disease, leading to publication bias. Finally the quality of the studies included in the review is generally poor. This calls for standardized diagnostic and survey methods to be used in future work as we progress towards developing the global atlas of podoconiosis[65].

This systematic review provides a comprehensive global overview of the prevalence of podoconiosis. We found podoconiosis to be a common public health problem affecting underprivileged areas of the tropics with particularly high prevalence in the African Region. Despite the methodological limitations of most of the studies conducted, the evidence cumulatively indicates that podoconiosis remains an important and unrecognized public health problem in this region. As noted by Hotez recently, podoconiosis is a clear example of a disease of enormous importance which has been neglected by the global community [66]. To address this neglect, control and prevention interventions must be expanded in countries recognized to be endemic. Standardized population based surveys will be vital in clarifying the global distribution of podoconiosis and further accelerating the elimination of this preventable and treatable disease.

## Supporting information

S1 Checklist PRISMA checklist. (DOC)

S1 Text Supplementary file: Search strategy and quality of included studies. (DOCX)
