# Supplementary material for: Global epidemiology of podoconiosis: A systematic review
Source: PLoS Negl Trop Dis. 2018 Mar 1;12(3):e0006324. doi: 10.1371/journal.pntd.0006324 (PMC5849362; doi:10.1371/journal.pntd.0006324)
Supplement: S1 Text — (DOCX) [file pntd.0006324.s002.docx]

**Global epidemiology of podoconiosis: a systematic review**

**Supplementary File**

1. **Search strategy**

The following list shows steps in the key term strategy, using MEDLINE, resulting in the total number of abstracts screened on February 14, 2017.

| **Table A. Search approach used in MEDLINE** | | |
| --- | --- | --- |
| **SN** | **Searches** | **Results** |
| 1 | elephantiasis | 3705 |
| 2 | podoconiosis | 3725 |
| 3 | Non filrarial | 3043 |
| 4 | Mossy foot | 3712 |
| 6 | 1 or 2 or 3 or 4 | 5446 |
| 7 | Incidence/ | 2,527,133 |
| 8 | Prevalence/ | 2,369,189 |
| 9 | Epidemiology/ | 2,128,258 |
| 10 | Public Health/ | 7,213,551 |
| 11 | Population/ | 1,566,318 |
| 13 | 7 or 8 or 9 or 10 or 11 | 8,453,935 |
| 14 | 6 and 13 | 2594 |

Syntax used for the search

(((("elephantiasis"[MeSH Terms] OR "elephantiasis"[All Fields] OR ("mossy"[All Fields] AND "foot"[All Fields]) OR "mossy foot"[All Fields]) OR (non[All Fields] AND filarial[All Fields])) OR ("elephantiasis"[MeSH Terms] OR "elephantiasis"[All Fields] OR "podoconiosis"[All Fields])) OR ("elephantiasis"[MeSH Terms] OR "elephantiasis"[All Fields])) AND ((((("population"[MeSH Terms] OR "population"[All Fields] OR "population groups"[MeSH Terms] OR ("population"[All Fields] AND "groups"[All Fields]) OR "population groups"[All Fields]) OR ("public health"[MeSH Terms] OR ("public"[All Fields] AND "health"[All Fields]) OR "public health"[All Fields])) OR ("epidemiology"[Subheading] OR "epidemiology"[All Fields] OR "epidemiology"[MeSH Terms])) OR ("epidemiology"[Subheading] OR "epidemiology"[All Fields] OR "prevalence"[All Fields] OR "prevalence"[MeSH Terms])) OR ("epidemiology"[Subheading] OR "epidemiology"[All Fields] OR "incidence"[All Fields] OR "incidence"[MeSH Terms]))

1. **Quality assessment of included studies**

The table summarises the quality assessment of all included reports using the following quality assessment framework.

Scoring matrix for 4 quality assessment elements:

**A. Sampling frame defined**

0. No information beyond overall population type (eg “schools” or “households”)

1. General information on sampling frame and procedures

2. Explicit details of procedures reported

**B. Response rate**

0. Not recorded/reported

1. Reported and under 65%

2. Reported and 65% or above

**C. Quality of podoconiosis assessment**

0. No detail provided

1. General information on assessment methods

2. Detailed description on assessment methods and inclusion of diagnostic criteria

**D. Statistical analysis**

0. Only overall prevalence reported

1. Age-specific prevalence reported

2. Prevalence by age and other criteria (eg geographic, socio-demographic) reported

| Table B. Quality of studies included in the systematic review | | | | | | |
| --- | --- | --- | --- | --- | --- | --- |
| SN | Citation | A.  Sampling frame defined | B.  Response rate | C.  Quality of podoconiosis assessment | D.  Statistical analysis methods | Total quality score out of 8 |
| 1 | Price EW. Endemic elephantiasis of the lower legs in Rwanda and Burundi. Trop Geogr Med. 1976; 28 283-90. | 1 | 0 | 1 | 1 | 3 |
| 2 | Wanji S, Tendongfor N, Esum M, Che JN, Mand S, Tanga Mbi C, et al. Elephantiasis of non-filarial origin (podoconiosis) in the highlands of north-western Cameroon. Ann Trop Med Parasitol. 2008; 102(6):529-40. | 0 | 0 | 1 | 2 | 3 |
| 3 | Cho-Ngwa F, Amambua AN, Ambele MA, Titanji VPK. Evidence for the exacerbation of lymphedema of geochemical origin, podoconiosis, by onchocerciasis. Journal of Infection and Public Health 2009; 2:198-203. | 0 | 0 | 2 | 2 | 4 |
| 4 | Wanji S, Kengne-Ouafo JA, Datchoua-Poutcheu FR, Njouendou AJ, Tayong DB, Sofeu-Feugaing DD, et al. Detecting and staging podoconiosis cases in North West Cameroon: positive predictive value of clinical screening of patients by community health workers and researchers. BMC Public Health. 2016; 16:997. | 2 | 2 | 1 | 0 | 5 |
| 5 | Deribe K, Andrew AB, Cano J, Jelil A, Fru-Cho J, Raphael A, et al. Mapping the geographical distribution of podoconiosis in Cameroon using parasitological, serological, and clinical evidence to exclude other causes of lymphedema. PLoS Negl Trop Dis. 2017 Inpress | 2 | 2 | 2 | 2 | 8 |
| 6 | Price E. Podoconiosis:Non-filarial Elephantiasis. Oxford Medical Publications, Oxford, UK. 1990. | 0 | 0 | 1 | 1 | 2 |
| 7 | Bekele K, Deribe K, Amberbir T, Tadele G, Davey G, Samuel A. Burden assessment of podoconiosis in Wayu Tuka woreda, east Wollega zone, western Ethiopia: a community-based cross-sectional study. BMJ Open. 2016;6(9):e012308. | 2 | 2 | 1 | 2 | 7 |
| 8 | Deribe K, Brooker SJ, Pullan RL, Sime H, Gebretsadik A, Assefa A, et al. Epidemiology and individual, household and geographical risk factors of podoconiosis in Ethiopia: results from the first nationwide mapping. Am J Trop Med Hyg. 2015:148–58. | 2 | 2 | 2 | 2 | 8 |
| 9 | Tekola Ayele F, Alemu G, Davey G, Ahrens C. Community-based survey of podoconiosis in Bedele Zuria woreda, southwest Ethiopia. Int Health. 2013; 5(2):119-25. | 2 | 1 | 2 | 2 | 7 |
| 10 | Molla YB, Tomczyk S, Amberbir T, Tamiru A, Davey G. Podoconiosis in East and West Gojam zones, Northern Ethiopia. PLoS Negl Trop Dis. 2012; 6(7):e1744. | 2 | 1 | 2 | 2 | 7 |
| 11 | Geshere Oli G, Tekola Ayele F, Petros B. Parasitological, serological, and clinical evidence for high prevalence of podoconiosis (non-filarial elephantiasis) in Midakegn district, central Ethiopia. Trop Med Int Health. 2012; 17(6):722-6. doi: 10.1111/j.1365-3156.2012.02978. | 2 | 0 | 2 | 2 | 6 |
| 12 | Alemu G, Tekola Ayele F, Daniel T, Ahrens C, Davey G. Burden of podoconiosis in poor rural communities in Gulliso woreda, West Ethiopia. PLoS Negl Trop Dis. 2011; 5(6):e1184. | 2 | 0 | 1 | 2 | 5 |
| 13 | Desta K, Ashine M, Davey G. Prevalence of podoconiosis (endemic non-filarial elephantiasis) in Wolaitta, Southern Ethiopia Tropical Doctor. 2003; 32:217-20. | 2 | 0 | 1 | 2 | 5 |
| 14 | Birrie H, Balcha F, Jemaneh L. Elephantiasis in Pawe settlement area: podoconiosis or Bancroftian filariasis? Ethiopian Medical Journal. 1997; 35:245-50. | 2 | 0 | 2 | 2 | 6 |
| 15 | Frommel D, Ayranci B, Pfeifer HR, Sanchez A, Frommel A, Mengistu G. Podoconiosis in the Ethiopian Rift Valley. Role of beryllium and zirconium. Trop Geogr Med 1993; 45(4):165-7. | 2 | 0 | 2 | 2 | 6 |
| 16 | Kloos H, Kello AB, Addus A. Podoconiosis (endemic non-filarial elephantiasis) in two resettlement schemes in western Ethiopia. Tropical Doctor. 1992; 22:109-12. | 2 | 1 | 2 | 1 | 6 |
| 17 | Mengistu G, Humber D, Ersumo M, Mamo T. High prevalence of elephantiasis and cutaneous leishmaniasis in Ocholo, south-west Ethiopia. . Ethiopian Medical Journal. 1987; 25:203-7. | 2 | 0 | 1 | 1 | 4 |
| 18 | Price EW. The relationship between endemic elephantiasis of the lower legs and the local soils and climate: A study in Wollamo District, Southern Ethiopia. Trop Geogr Med 1974; 26(3):225-30. | 1 | 1 | 1 | 0 | 3 |
| 19 | Oomen AP. Studies on elephantiasis of the legs in Ethiopia. Trop Geogr Med. 1969; 1969 (21):3. | 1 | 0 | 1 | 1 | 3 |
| 20 | Crivelli PE. Non-filarial elephantiasis in Nyambene range: a geochemical disease. East Afr Med J. 1986; 63(3):191-4. | 1 | 0 | 2 | 0 | 3 |
| 21 | Muli J, Gachohi J, Kagai J. Soil iron and aluminium concentrations and feet hygiene as possible predictors of Podoconiosis occurrence in Kenya. PLoS Negl Trop Dis. 2017; 11(8):e0005864. | 1 | 2 | 2 | 1 | 6 |
| 22 | Ruiz L, Campo E, Corachan M. Elephantiasis in Sao Tome and Principe. Acta Trop. 1994; 57(1):29-34. | 2 | 2 | 2 | 0 | 6 |
| 23 | Onapa AW, Simonsen PE, Pedersen EM. Non-filarial elephantiasis in the Mt. Elgon area (Kapchorwa District) of Uganda. Acta Trop. 2001; 78(2):171-6. | 2 | 2 | 2 | 2 | 8 |
| 24 | Kihembo C, Masiira B, Lali WZ, Matwale GK, Matovu JKB, Kaharuza F, et al. Risk Factors for Podoconiosis: Kamwenge District, Western Uganda, September 2015. Am J Trop Med Hyg. 2017; 96(6):1490-6. | 2 | 0 | 2 | 2 | 6 |
| 25 | De Meira MTV, Somoes TS, Nogueira JFP. On the existence of mossy foot on the island of St Nicolau (Cape Verde). Ann Inst Med Trop (Lisbon) 1947; 4:269-79. | 2 | 0 | 2 | 0 | 4 |
| 26 | Russel S, Rao CK, Rao CK. Prevalence of nonfilarial elephantiasis in selected towns in India. The Journal of communicable diseases. 1983; 15(3):216-8. PubMed PMID: 6672090. | 2 | 0 | 2 | 0 | 4 |
| 27 | Jordan P, Hope Trant M, Laurie W. Non-Bancroftian Elephantiasis in Tanganyika. British Medical Journal 1956;28:209-10. |  |  |  |  |  |
|  |  | 0 | 0 | 1 | 0 | 1 |

| **Table C. Sources of case report and presence records of podoconiosis** | | | |
| --- | --- | --- | --- |
| **Study, study or publication year** | | **Country** | **Reports** |
| Africa | |  |  |
|  | Price & Henderson,1980 [1] | Cameroon | 130 cases |
|  | Corachan et al.,1988[2] | Equatorial Guinea | 2 cases |
|  | Morrone et al., 2011[3] | Ethiopia | 18 cases |
|  | Cohen,1960 [4] | Ethiopia and Kenya | 32 (13 Kenya and 19 Ethiopia) |
|  | Clark,1948[5] | Kenya | 200cases |
|  | Jordan et al.,1956 [6] | Tanzania | 74 cases |
|  | de Lalla et al.,1988 [7] | Tanzania | 30 cases |
|  | Eid et al.,2016[8] | Tanzania | 1 case |
|  | Lowenthal,1934[9] | Uganda | 11 cases |
|  | Dwek et al.,2015[10] | Uganda | 3 cases |
|  | Price,1990[11] | Sudan | 28 cases |
|  | Price & Bailey,1884[12] | Sudan | 10 cases |
|  | Price,1990 [11] | Nigeria | Reported presence |
| Asia | |  |  |
|  | Price,1990[11] | Sri Lanka | Reported presence |
|  | Kalra, 1976[13] | India | 6 cases |
|  | Price,1990[11] | Indonesia | Reported presence |
| Latin America | |  |  |
|  | Price,1990[11, 14] | Mexico | Reported presence |
|  | Price,1990[11, 15] | Guatemala | Reported presence |
|  | Price,1990[11] | Ecuador | Reported presence |
|  | Tada & Marsden, 1993[16] | Brazil | 1 case |
|  | Price,1990[11] | Suriname | Reported presence |
|  | Price,1990[11] | French Guiana | Reported presence |
|  | Price,1990[11] | Colombia | Reported presence |
|  | Price,1975[11] | El Salvador | Reported presence |
|  | Price,1990[11] | Costa Rica | Reported presence |
|  | Lowenthal,1934[9] | Honduras | Reported presence |
|  | Lowenthal,1934[9] | Peru | Reported presence |

**References**

1. Price EW, Henderson WJ. Endemic elephantiasis of the lower legs in the United Cameroon Republic. Trop Geogr Med 1981;33(1):23-9.

2. Corachan M, Tura JM, Campo E, Soley M, Traveria A. Podoconiosis in Aequatorial Guinea. Report of two cases from different geological environments. Trop Geogr Med 1988;40(4):359-64.

3. Morrone A, Padovese V, Dassoni F, Pajno MC, Marrone R, Franco G, et al. Podoconiosis: an experience from Tigray, Northern Ethiopia. J Am Acad Dermatol. 2011;65(1):214-5.

4. Cohen LB. Idiopathic lymphoedema of Ethiopia and Kenya. East Afr Med J. 1960;37:53-74.

5. Clark M. Lymphostatic Verrucosis in the Fort Hall District of Kenya. Trans R Soc Trop Med Hyg. 1948;42(3):287-90.

6. Jordan P, Hope Trant M, Laurie W. Non-Bancroftian Elephantiasis in Tanganyika. British Medical Journal 1956;28:209-10.

7. de Lalla F, Zanoni P, Lunetta Q, Moltrasio G. Endemic non-filarial elephantiasis in Iringa District, Tanzania: a study of 30 patients. . Trans R Soc Trop Med Hyg 1988;82( 6):895-7.

8. Eid R, Sharma D, Smock W. Images in Clinical Tropical Medicine Podoconiosis in Rural Tanzania. Am J Trop Med Hyg. 2016;95(1):1.

9. Lowenthal LJA. On the probable inclusion of several diseases in the title "mossy" foot. Ann Trop Med Parasitol 1934;28:47-57.

10. Dwek P, Yuan KL, Wafer M, Cherniak W, Pace R, Malhamé I, et al. Case Report and Literature Review: Podoconiosis in Southwestern Uganda. International Journal of TROPICAL DISEASE & Health 2015;9(3):1-7.

11. Price E. Podoconiosis:Non-filarial Elephantiasis. Oxford Medical Publications, Oxford, UK. 1990.

12. Price EW, Bailey D. Environmental factors in the etiology of endemic elephantiasis of the lower legs in tropical Africa. . Trop Geogr Med 1984;36(1):1-5.

13. Kalra NL. Non-fllarial elephantiasis in Bikaner, Rajasthan. Journal of Communicable Diseases 1976;8 (4):337-40.

14. Mazzotti L. Pseudo-Leprosy of Robles in Mexico. Medicina 1941;21(383):103-7.

15. R R. Pseudo-Leprosy or Punudos, an Unclassed Disease of Guatemala. Bull Acad Med. 1927;(23):776-80.

16. Tada MS, Marsden PD. Probable podoconiosis in Brasilia. Rev Soc Bras Med Trop. 1993;26(4):255.
